# A Symmetry-Based Perspective Correction Method for High-Speed Deformation Analysis of Circular Blast-Loaded Plates

**DOI:** 10.3390/ma19132928

**Published:** 2026-07-07

**Authors:** Edison Shehu, Georgios Kechagiadakis, Bachir Belkassem, Andrea Manes, Frederik Coghe, David Lecompte

**Affiliations:** 1 Politecnico di Milano, Dipartimento di Meccanica, Via La Masa, 1, 20156 Milan, Italy; 2 Structures and Effects of Explosions Department, Royal Military Academy, Av. de la Renaissance 30, B-1000 Brussels, Belgium; 3 Department of Weapon Systems and Ballistics, Royal Military Academy, Av. de la Renaissance 30, B-1000 Brussels, Belgium

**Keywords:** blast loading, thin-walled structures, aluminum alloy, structural response, optical correction, symmetry

## Abstract

The objective of this study is to recover the transient out-of-plane displacement field of clamped circular plates subjected to blast loading using a single high-speed camera, as a low-cost alternative to stereo Digital Image Correlation (DIC) for the specific class of axisymmetrical structural responses of circular plates. The dynamic response of thin metal plates to blast loading is a fundamental problem in protective structural design, traditionally investigated through DIC. Although it provides full-field displacement measurements with high spatial resolution, it requires stereo camera arrangements, controlled illumination, speckle pattern preparation, and elaborate calibration procedures that significantly increase experimental cost and complexity. This study introduces a monocular optical method applicable to axisymmetrically defined material testing applications, such as the response of circularly supported isotropic plates under a uniform impulsive load, to recover the transient out-of-plane displacement field without using DIC. Clamped circular aluminum plates are subjected to blast loading generated by PG-3 charges of variable mass detonated at the closed end of a shock tube, with the exposed face matching the tube cross-section so as to enforce axisymmetric pressure load. A diametral reference line marked on the rear face of each specimen was recorded by a single high-speed camera, and a perspective correction derived from the axisymmetric deformed geometry was then applied to reconstruct the time-resolved displacement profile along the diameter. The permanent post-test deformed shape of each plate was subsequently digitized through 3D scanning and used as ground truth to validate the optical reconstruction. The reconstructed profiles closely matched the scans: for the conventional responses the root-mean-square error was 1.251 mm with a normalized mean residual of 6.57% (Case A) and 1.793 mm (9.20%, Case B), while for the anomalous counterintuitive response it was 1.043 mm (14.93%, Case C). Symmetry can thus be exploited as an active measurement principle to obtain quantitative blast-response data with substantially reduced experimental burden and without specialized stereo-optical instrumentation.

## 1. Introduction

Thin metallic plates subjected to impulsive and blast loading represent a classical problem in structural impact and protective design. Their response combines transient pressure loading, large inelastic deformation, membrane stretching, strain-rate effects and boundary-condition sensitivity, and have been used for decades as benchmarks for both analytical models and numerical simulation. The seminal two-part review by Nurick and Martin [1,2] established the theoretical and experimental framework still used to interpret thin-plate blast response, and the three failure modes they identified as large ductile deformation (Mode I), tensile tearing (Mode II) and transverse shear (Mode III) remain the standard classification. Subsequent experimental work on clamped circular plates by Teeling-Smith and Nurick [3] and analytical extensions by Wen [4] for work-hardening materials further refined the prediction of permanent deflection and rupture thresholds. A 25-year update by Yuen, Nurick and co-workers [5] confirmed that, despite significant advances in instrumentation and numerical methods, monolithic clamped plates remain a key reference configuration. Among plate geometries, the clamped circular plate is particularly attractive because, under a centered and approximately uniform impulse, the dominant out-of-plane displacement field is axisymmetric and can be expressed as w=w(r,t). This is not merely a modeling convenience: when the loading and clamping are themselves axisymmetric, radial symmetry becomes a physically grounded constraint that can be exploited at the measurement stage. The use of circular explosion-driven shock tubes (EDST) is well suited to this configuration because the specimen is mounted at the end of a circular tube and the exposed area matches the tube cross-section, producing a centered and quasi-uniform pressure pulse free of other surface reflections [6]. Such experimental configurations have been used extensively to investigate the dynamic response of aluminum and steel plates [7], and recent experimental and numerical studies have continued to characterize their ductile failure, the influence of the negative loading phase and the counterintuitive (reversed) deformation observed at low impulses [8,9,10]. Beyond idealized laboratory specimens, the same impulsive-loading mechanics underpin the protective design of a broad range of engineering structures, including metallic and fiber-composite panels for naval and defense platforms [11], reinforced-concrete columns subjected to localized blast [12], and energy-absorbing structural components engineered to dissipate impact and explosive loading [13], where reliable full-field deformation data remain essential for design and validation.

Accurate characterization of transient deformation during blast loading remains, however, a measurement-side challenge. Local sensors such as strain gauges, accelerometers, and displacement transducers provide time histories only at discrete locations and can be intrusive or vulnerable in the harsh blast environment. For this reason, optical full-field techniques have become central in dynamic testing. Digital Image Correlation (DIC), in particular two-dimensional and stereoscopic three-dimensional DIC, has emerged as the de facto standard for extracting full-field displacement and strain from high-speed image sequences; its theoretical and practical foundations are extensively documented in the literature [14,15].

The application of stereo-DIC to blast-loaded plates has produced rich validation datasets. Tiwari et al. [16] pioneered the use of high-speed three-dimensional image correlation for transient plate deformation during blast loading, and Spranghers and co-workers reported full-field measurements of aluminum plates under free-air blast [17] and complementary numerical–experimental studies on the same configuration [18]. More recently, high-speed stereo-DIC has been combined with explosive-driven shock tube loading to characterize aluminum plates under blast and combined fragment–blast loading [6,19]. These works demonstrate the strength of stereo-DIC for producing spatially resolved transient deformation fields suitable for finite-element validation and inverse material identification. They also expose its practical cost: stereo-DIC requires two synchronized high-speed cameras with accurate stereo calibration, intense and stable illumination, a high-contrast speckle pattern that survives the event, and unrestricted optical access. These conditions are not always available in destructive close-range blast experiments. Moreover, the cost of a high-speed camera with sufficient resolution at frame rates relevant to the timescales of highly dynamic events like blast loading can be prohibitively expensive, ranging from several tens to a few hundreds of thousands of USD.

Despite these advances, the recent literature on blast-loaded plates has continued to rely either on discrete local sensors or on dual-camera stereo-DIC, and no low-cost measurement strategy has been proposed that explicitly exploits the axisymmetry of the response to recover quantitative deformation fields from a single camera. When the underlying mechanical response is known to be predominantly axisymmetric, a full stereo reconstruction provides more generality than the measurement objective strictly requires. This observation motivates the use of constrained monocular optical methods. The geometric link between a three-dimensional scene and its image-plane projection is governed by projective geometry [20], and flexible planar-target camera calibration techniques, established by Zhang [21] and complemented by methods exploiting circular features [22], make perspective correction accessible without elaborate metrology rigs. Combined with strong prior knowledge of specimen geometry and deformation mode, a single high-speed camera can yield quantitative out-of-plane displacement information that would otherwise be ambiguous in a general three-dimensional scene.

The present work builds on this principle and proposes a monocular, symmetry-based perspective correction method for the high-speed deformation analysis of blast-loaded clamped circular plates. A marked diametral generator on the rear face of the plate is tracked in a single high-speed sequence; its apparent image-plane curve is converted into a one-dimensional out-of-plane displacement profile through a calibrated rational projective mapping, and an equivalent axisymmetric deformation field can be reconstructed by radial revolution. Permanent post-test three-dimensional scan data provides an independent ground truth. The method is not intended as a universal replacement for stereo-DIC, which remains preferable when general non-axisymmetric full-field measurements are required; rather, it targets the specific but practically relevant class of experiments in which geometry, loading and expected response justify an axisymmetric reduction. In these cases, symmetry is treated not as a post-processing assumption but as an active part of the measurement strategy itself. The main contribution of this work is therefore methodological: for the broad and practically relevant class of axisymmetric blast and impact problems, it shows that quantitative transient out-of-plane fields can be recovered with a single high-speed camera and a calibrated projective model, removing the need for the synchronized dual-camera hardware, stereo calibration and full speckle coverage required by the stereo-DIC studies discussed above [16,17,18,19] and thereby lowering the cost and complexity of full-field blast diagnostics.

## 2. Materials and Methods

### 2.1. Experimental Setup

**Figure 1:**
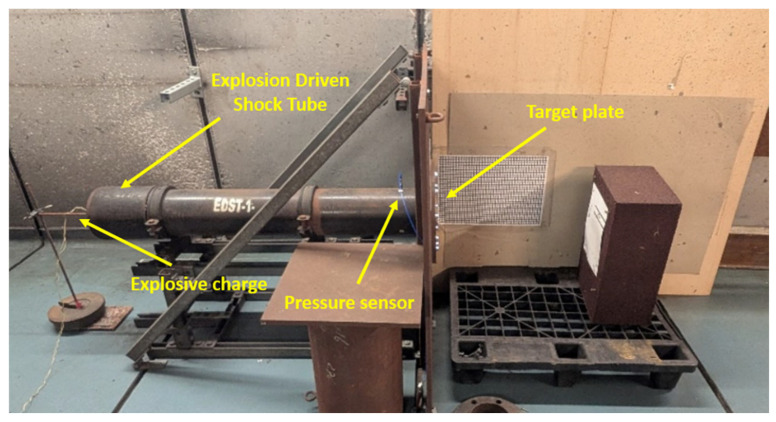
Experimental configuration of the explosion-driven shock tube (EDST) test. The blast wave is generated by an explosive charge at the closed end of the tube and propagates toward the clamped circular target plate. A pressure sensor is positioned along the tube to monitor the loading event.

The experiments were conducted under laboratory-controlled conditions using an explosion-driven shock tube (EDST) facility located at the Royal Military Academy in Brussels, Belgium. The EDST consists of a primary cylindrical steel tube of 1200 mm length, 4.5 mm wall thickness and 168.2 mm inner diameter. The entrance section is reinforced over its first 200 mm by a concentric outer steel tube with a 4.5 mm wall thickness and a 193.7 mm inner diameter, providing additional structural rigidity at the charge-end. A small spherical PG-3 plastic-explosive charge is detonated at the center of the tube entrance, generating a planar blast wave that propagates along the tube axis. The target plate is clamped at the opposite end of the tube; the clamping fixture exposes a circular area of 150 mm diameter to the blast loading while the surrounding annular region remains clamped and can be considered rigid for the purposes of the present analysis. The experimental configuration, including the shock tube geometry, charge position, pressure sensor location, and target plate assembly, is illustrated in Figure 1.

The specimens are aluminum plates made of AA2024-T3 alloy 0.5 mm thick manufactured via cold-rolling and clamped by a rigid annular fixture. This aluminum alloy is a high-strength, age-hardenable aluminum–copper–magnesium alloy of the 2000 series (nominal composition Al–4.4Cu–1.5Mg–0.6Mn wt%) that is widely used in aerospace structures for its high strength-to-weight ratio and damage tolerance; the T3 temper denotes solution heat treatment, cold working and natural aging. Its nominal physical and quasi-static mechanical properties are summarized in Table 1 [23,24].

**Figure 2:**
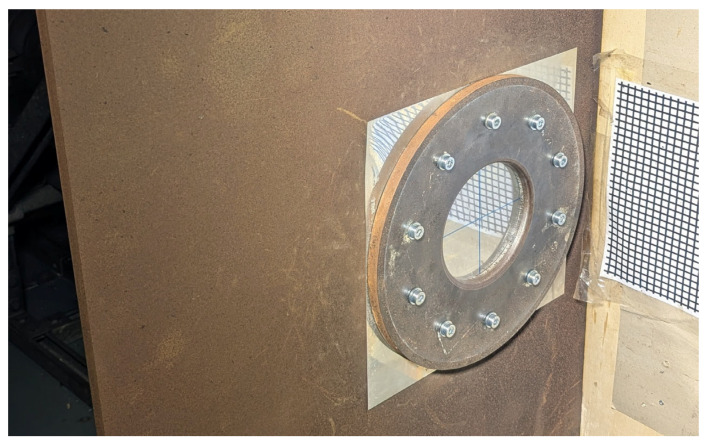
Detail of the clamped circular target plate. The annular fixture constrains the specimen around the exposed circular region. The rear surface remains visible to the high-speed camera and carries the reference marks used for video processing.

The fixture constrains the plate around the exposed circular area and is tightened using a bolted connection, as shown in Figure 2. The exposed region corresponds to the circular shock tube opening, so that the loading is axisymmetrically applied. The rear face of the plate is visible to the high-speed camera and is marked before each test. The diameter of the exposed circle is 150 mm.

Three representative tests are considered in this paper. Cases A and B correspond to a conventional response, where the deformed shape remains consistent with the expected behavior of a clamped circular plate. Case C corresponds to an anomalous response, where the high-speed sequence and the post-test specimen inspection reveals a non-standard deformation pattern. Case C constitutes a more demanding validation scenario: unlike the monotonically increasing displacement profile expected for a clamped circular plate, the measured diameter exhibits local extrema, producing a spatially complex displacement curve that must be accurately reconstructed by the method. In addition, the scan comparison serves to assess whether the global deformation departs from the axisymmetric assumption that underlies the proposed approach. The three scenarios are summarized in Table 2. The three charges were selected in order to induce different response regimes that the method is intended to handle rather than to reproduce a single nominal condition: the larger charges (Cases A and B, 3.7 and 3.9 g) produce the conventional, membrane-dominated ductile dome, whereas the smaller charge (Case C, 2.0 g) lies in the low-impulse regime in which the reversed, counterintuitive response can develop. The three cases are therefore representative of the qualitatively different behaviors (conventional axisymmetric and anomalous non-axisymmetric) against which the robustness of the reconstruction must be assessed, whereas a broader parametric mapping of the response as a function of charge mass lies beyond the scope of the present methodological study.

### 2.2. Specimen Marking, Image Acquisition and Point Layout

A vertical diametral line is marked on the rear face of each plate. A horizontal reference mark passing through the center is also present. The vertical line is the main measurement feature: it connects two visible edge anchors located at the top and bottom of the blasted circular area. The horizontal mark is used to identify the center and to verify the orientation of the plate in the image sequence.

The rear face of the specimen is recorded during the blast event using a Photron FASTCAM SA-X2 (type 1080K-M4) high-speed camera (Photron Ltd., Tokyo, Japan), operated at 60,000 fps with a resolution of 384  ×  468 pixels. The acquired frames are processed as grayscale images, in which the marked vertical line appears as a high-contrast dark feature against the plate surface. The line is discretized into N = 21 tracking stations, labeled $p_{1}$ to $p_{21}$ from the top anchor to the bottom anchor. The first and last stations, $p_{1}$ and $p_{21}$, are treated as boundary anchors. The internal stations $p_{2}$ to $p_{20}$ are used to reconstruct the displacement of the marked diameter.

The nominal physical coordinate $x_{i}$ assigned to each tracking station is reported in Equation (1).

(1) $$x_{i}=D\frac{i-1}{N-1},\;\;\;i=1,\ldots ,N$$

where D = 150 mm is the diameter of the blasted circular region and N = 21. Therefore, $p_{1}$, $p_{6}$, $p_{11}$, $p_{16}$ and $p_{21}$ correspond to 0, 37.5, 75, 112.5, and 150 mm, respectively. These five stations are used in the time-history plots because they provide a compact description of the boundary, quarter-span, center, three-quarter-span, and opposite boundary response.

### 2.3. Manual Initialization and Geometric Regularization

The tracking procedure begins with a manual initialization on the first frame. Four points are selected by the user:

1. The top anchor of the vertical diameter;
2. The center of the plate;
3. The bottom anchor of the vertical diameter;
4. A right-side reference point on the horizontal diameter.

The top and bottom anchors define the initial vertical baseline. The center and right-side points are used to check the orientation and scale of the visible circular area. The internal tracking points are then initialized along the top–bottom baseline and are regularized to be evenly spaced between the two anchors. This step is important because a manual point placement is never perfectly uniform. Without regularization, small spacing errors in the first frame would propagate into the final displacement profiles and into the comparison with the scan section.

Let $p_{top}$ and $p_{bottom}$ be the selected top and bottom anchor points in pixel coordinates. The initial position of ith point is computed as indicated in Equation (2).

(2) $$p_{i,0}=p_{top}+\frac{i-1}{N-1}\left(p_{bottom}-p_{top}\right),\;\;i=1,\ldots ,N$$

Thus, $p_{1}$ coincided with the top anchor, $p_{21}$ with the bottom anchor, and all internal points are evenly distributed along the reference diameter. This creates a consistent one-dimensional material coordinate along the marked diameter. The algorithm therefore tracks displacement histories at fixed nominal stations, not at arbitrary manually selected pixel positions.

### 2.4. Signed Perpendicular Pixel Offset

The out-of-plane measurement is implemented indirectly through the apparent displacement of the marked line in each frame. Therefore, the primary geometric operation is the conversion of the detected displacement of each tracking point into a signed perpendicular offset relative to the undeformed top–bottom baseline.

Let the top and bottom anchors in image coordinates be $p_{top}=\left(x_{T},y_{T}\right)$ and $p_{bottom}=\left(x_{B},y_{B}\right)$. The baseline vector v is defined in Equation (3):

(3) $$v=p_{bottom}-p_{top}=(x_{B}-x_{T},y_{B}-y_{T})=\left(dx,dy\right).$$

The length of the baseline vector L is reported in Equation (4):

(4) $$L=\sqrt{dx^{2}+dy^{2}}.$$

A unit tangent vector t along the reference diameter is defined as in Equation (5):

(5) $$t=\frac{v}{L}=\left(\frac{dx}{L},\frac{dy}{L}\right).$$

A normal direction in the image plane is obtained by rotating the tangent by 90 degrees. In the implemented convention, the non-normalized normal vector is defined as $h=\left(dy,-dx\right)$ and the corresponding unit normal n is defined as in Equation (6).

(6) $$n=\frac{h}{L}=\left(\frac{dy}{L},-\frac{dx}{L}\right).$$

For a tracked point $p_{i}\left(t\right)$ = ($x_{i}\left(t\right)$, $y_{i}\left(t\right)$) its signed perpendicular pixel offset is the scalar projection of $p_{i}\left(t\right)-p_{top}$ onto n, as reported in Equation (7):

(7) $$z_{px,i}\left(t\right)=\left[p_{i}\left(t\right)-p_{top}\right]\cdot n=\frac{\left[x_{i}\left(t\right)-x_{T}\right]dy-\left[y_{i}\left(t\right)-y_{T}\right]dx}{L}.$$

This expression is the two-dimensional signed distance from the undeformed reference diameter. Its sign is determined by the orientation of the chosen normal vector. The sign convention is kept fixed throughout the processing, so positive and negative displacements remain comparable between frames.

**Figure 3:**
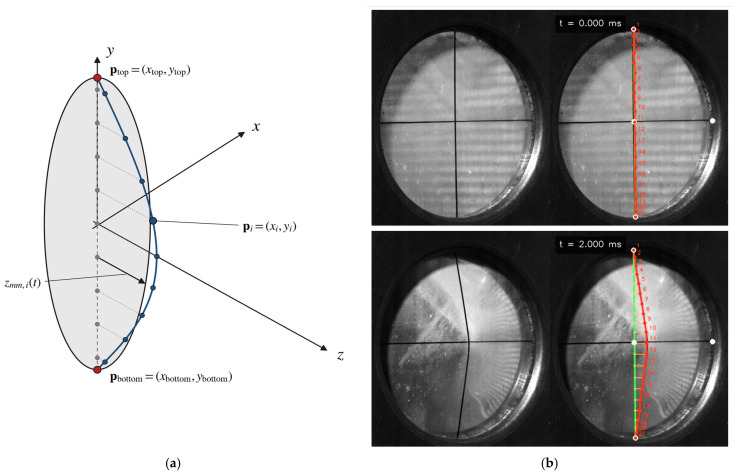
Geometric representation of the symmetry-based tracking method. The top and bottom anchors define the undeformed baseline. Internal points are tracked along the deformed marked line, and their signed perpendicular image-plane offset is converted into an out-of-plane displacement through a one-dimensional projective correction. (**a**) Geometric representation of the symmetry-based tracking method. (**b**) Two selected timeframes where the symmetry-based tracking algorithm was applied. Top and bottom show two different timesteps (Case B). Green line shows undeformed profile, red line shows deformed profile at t = 2 ms.

Equation (7) converts the two-dimensional image coordinates of each tracked point into a scalar signed perpendicular displacement relative to the undeformed reference diameter. Figure 3 provides a geometric illustration of this operation. The top and bottom anchor points define the instantaneous baseline from which all offsets are measured; they are identified in each frame and used to recompute the unit tangent vector **t** and its orthogonal unit normal **n**. The internal tracking points distributed along the marked line are shown at their deformed positions, and the dashed projection lines indicate the scalar projection of each point onto **n**, yielding the signed pixel offset, $z_{px,i}$. This quantity is subsequently converted into the physical out-of-plane displacement, $z_{mm,i}$, via the rational projective mapping described in Section 2.5. The values $z_{mm,i}$ are read over time. Since the signed offset is always computed relative to the current anchor-to-anchor baseline rather than relative to a fixed image axis, any small rigid-body rotation of the plate within the camera field of view is absorbed into the updated baseline and does not propagate into the out-of-plane displacement estimate.

### 2.5. Pixel-to-mm Rational Projective Correction

The signed pixel offset $z_{px,i}(t)$ is not yet a physical displacement. A constant pixel-to-mm scale would be insufficient because the camera views the target under perspective. A given physical displacement does not necessarily produce the same apparent shift in the pixels everywhere along the line of sight. To account for this effect while preserving a one-dimensional formulation, a rational projective mapping is used as reported in Equation (8) [20].

(8) $$z_{mm,i}\left(t\right)=\frac{az_{px,i}(t)}{1+bz_{px,i}(t)}$$

The coefficients a and b used in the present processing are reported in Table 3. The coefficient a sets the local millimeter-per-pixel scale near zero offset, while b introduces the perspective-induced rate of change in that scale with depth.

**Figure 4:**
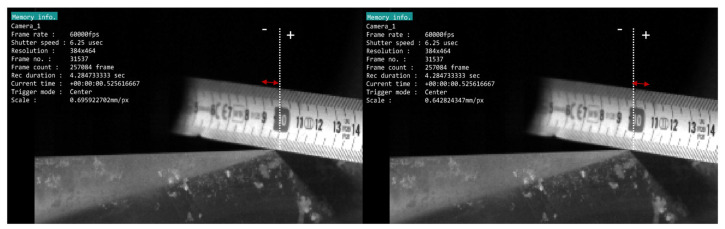
Perspective correction used in the proposed method. The apparent displacement of each tracked point is measured as a signed perpendicular pixel offset (red arrow) relative to the reference diameter and converted into millimeter by a calibrated rational one-dimensional mapping.

The two free parameters a and b are determined through a two-point calibration procedure. The proposed mapping is not an empirical interpolation law, but the one-dimensional expression of the projective relationship linking the plate’s physical out-of-plane displacement to its apparent displacement in the image plane. More details on the derivation of Equation (8) are given in Appendix A. The calibration target is placed at two known out-of-plane positions, $z_{mm}$ = +10 mm and $z_{mm}$ = −10 mm, relative to the reference plane, and the corresponding lateral scale (in mm/px) is measured directly from the high-speed camera acquisition software (Photron PFV4, version 4.6.1.0), yielding $S^{-}$ = 0.6959 mm/px and $S^{+}$ = 0.6428 mm/px, respectively, as reported in Figure 4.

Inverting the rational mapping, the secant scale at a given depth satisfies the linear relation $z_{\mathrm{mm}}/z_{\mathrm{px}}=a-b\cdot z_{\mathrm{mm}},$ so that the two measurements provide a 2 × 2 linear system whose closed-form solution is $a=(S^{-}+S^{+})/2$ and $b=(S^{+}-S^{-})/(2N),$ with N=10 mm. This yields a=0.66937 mm/px, representing the true lateral scale at the reference plane, and b=0.002655 px^−1^, quantifying the rate of perspective distortion with depth. Here b is defined with the sign convention that yields a positive value for a target approaching the camera, consistent with the value reported above.

The positive z-direction is defined as displacement towards the camera. Under perspective projection, a target moving towards the camera subtends a larger solid angle, so that a given physical displacement produces a larger apparent pixel excursion; equivalently, each pixel corresponds to a larger physical length (higher mm/px scale). This is consistent with $S^{-}$ > $S^{+}$: at $z_{mm}$ = +10 mm the scale is 0.6959 mm/px, whereas at $z_{mm}=-10$ mm it drops to 0.6428 mm/px. The positive value of b reflects this geometry: as $z_{\mathrm{px}}$ increases (plate approaching the camera), the denominator $1+bz_{\mathrm{px}}$ increases, reducing the effective mm/px conversion, in agreement with projective magnification.

The calibration requires only two physical positions in order to scale the spatial distortion induced by the perspective view. This simple solution is consistent with the projective geometry of a perspective camera observing a target that is displaced along an optical axis without the use of an optical model.

The coefficient b introduces a projective correction that modifies the effective scale as the apparent displacement increases. For small offsets, the denominator is close to one and the mapping behaves approximately linearly as reported in Equation (9):

(9) $$z_{mm,i}\left(t\right)\approx az_{px,i}\left(t\right),\;\;\mathrm{when}\;\left|bz_{px,i}\left(t\right)\right|\ll 1.$$

For larger offsets, the denominator changes the conversion factor and compensates for the perspective-induced shift in the apparent scale. This is why the mapping is rational rather than purely linear.

Therefore, the effective millimeter-per-pixel conversion is not constant. This is essential for the present setup because the reference line moves in a direction that is not parallel to the image plane. The mapping is interpreted as a calibrated one-dimensional correction valid for the chosen camera position and the marked diameter, not as a full three-dimensional camera reconstruction.

After initialization, each frame is processed using the previous frame as a predictor. For each point $p_{i}$ and each frame j, the algorithm performs the following operations:

1. It predicts the expected point location from the previous frame.
2. It defines a local search segment along the normal direction to the reference diameter.
3. It samples the grayscale intensity along that segment.
4. It identifies the most likely position of the dark marked line inside the local search interval.
5. It updates the point coordinate $p_{i}(t_{j})$.
6. It computes $z_{px,i}(t_{j})$ using the signed perpendicular projection.
7. It converts $z_{px,i}(t_{j})$ into $z_{mm,i}(t_{j})$ using the rational mapping.

The search is performed along the normal direction rather than in a two-dimensional region because the expected physical out-of-plane displacement of the marked line is mainly lateral in the image. This made the tracking faster and more robust. It also avoids unnecessary degrees of freedom: the method does not attempt to track arbitrary two-dimensional texture variation, only the apparent displacement of a known material line.

### 2.6. Outlier Rejection and Profile Consistency

High-speed blast images are difficult to process. The marked line can be partially blurred, locally hidden by lighting changes, intersected by the horizontal reference mark, or disturbed by reflections and contrast variations. Therefore, the raw point detections need to be reviewed or validated, ensuring they represent reality and are devoid of optical artifacts. The consistency of the implemented algorithm is enforced across three distinct layers.

First, the points are constrained by their initial one-dimensional ordering along the diameter. The algorithm does not allow the point sequence to become physically scrambled. This prevents a local detection error from displacing a point past its neighbors along the diameter.

Second, temporal consistency is enforced. A newly detected displacement is compared with the previous value at the same point. If the frame-to-frame jump is incompatible with the neighboring histories or with the expected smooth evolution, the value is flagged as a potential outlier.

Third, the spatial consistency is checked along the profile. At a given time, the displacement of point $p_{i}$ is compared with the local trend defined by points $p_{i-1}$ and $p_{i+1}$. Isolated spikes are suppressed because they are more likely to be tracking artifacts than real localized deformation of the plate over one station spacing.

The center point $p_{11}$ requires particular attention because it is positioned close to the intersection between the vertical and horizontal marks. When the horizontal reference line remains visible, the local image gradient at the intersection can pull the center detection onto the horizontal mark rather than the vertical reference line. Therefore, a conditional stabilization algorithm is used in order to correct isolated non-physical jumps of the points by comparing the displacement of the pixels around the center point with the neighboring pixels along the vertical reference line.

The result of the tracking stage is the displacement matrix defined in Equation (10):

(10) $$Z=\left[z_{mm,i}\left(t_{j}\right)\right]$$

with size T × N, where T is the number of processed frames and N = 21 is the number of tracking points. Each row of **Z** is an instantaneous diametral profile. Each column is the displacement history of a selected material station. To support reproducibility, the tracking pipeline is summarized here with its control parameters. After grayscale conversion, CLAHE equalization (clip 2.0, 8 × 8 tiles) and a 3 × 3 Gaussian blur, each of the N = 21 evenly paced stations is tracked along the normal to the reference axis within a ±14 px search window (0.25 px step, bilinear sampling, intensity averaged over a ±3 px tangential strip). The line is located at the minimum of an energy functional combining a dark-line intensity term, an edge term and a frame-to-frame jump penalty (weights 1.0, 0.15 and 0.45) and refined to sub-pixel accuracy by a parabolic fit; no temporal smoothing is applied to the histories. The two edge anchors $p_{1}$ and $p_{21}$ are held fixed at the clamped boundary. A point is corrected as an outlier only when it deviates from both its spatial predictor (a quadratic fit of the four neighbors) and its previous-frame value by more than max (1.75 mm, 2.75$\sigma_{local}$) and is then replaced by a 0.70/0.30 blend of the spatial and temporal predictors. Manual intervention occurs only at time zero, on the first frame, to verify that the stations are correctly located and evenly spaced along the marked diameter; the tracking then runs automatically over the whole sequence.

### 2.7. Time Synchronization and Selected Histories

The high-speed video provides a time vector t_j_ associated with the frame rate. In the present processing, the displacement histories are reported up to t = 40 ms for the selected point-history plots. This interval captures the rapid transient event and the subsequent transition towards a residual deformation level.

The boundary points $p_{1}$ and $p_{21}$ are expected to remain close to zero displacement because they correspond to the constrained edge anchors. The internal points describe the evolution of the deformed profile. The center point $p_{11}$ is particularly important because it is expected to show the largest displacement in a conventional axisymmetric dome-like response.

### 2.8. Post-Test 3D Scan Validation

**Figure 5:**
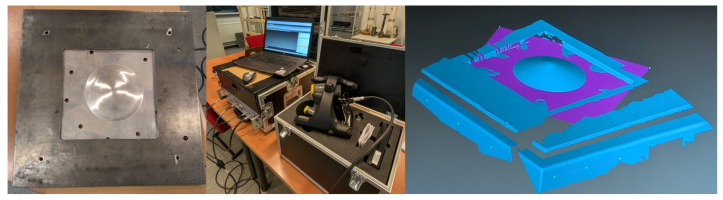
Overview of the 3D scan acquisition workflow. (**Left**): The blasted aluminum plate clamped within the steel frame used as a reference surface during scanning. (**Center**): The K-Scan optical tracking system and data acquisition hardware. (**Right**): The reconstructed point cloud of the deformed plate surface.

The post-test surface geometry was acquired using a Metris/Nikon Metrology ModelMaker MMDx handheld 3D laser scanner, integrated within a K-Scan/Krypton optical tracking system. The instrument consists of a manually operated laser scanning head whose position and orientation are tracked by an external optical localizer, enabling the acquired surface points to be registered within a common three-dimensional reference frame. To hold each blasted plate in a known, repeatable orientation during acquisition, a steel clamping frame was mounted on both faces of the specimen, providing a flat reference surface relative to which the deformed geometry was registered. During the acquisition, the scanner was swept over the deformed plate while maintaining the appropriate stand-off distance from the specimen surface. The workflow of the data acquisition procedure is illustrated in Figure 5.

The point cloud was processed to reconstruct the permanent out-of-plane deformation of the plate. From each scanned surface, a diametral cross-section is extracted along the same line used in the video processing, yielding a set of in-plane coordinates paired with the corresponding out-of-plane displacement values. This scan section serves as an independent reference for permanent deformation.

Because the scanner captures surface points beyond the blasted area, the raw coordinate extrema do not coincide with the physical boundary of the deformed region. The useful diameter is therefore delimited by the two sharp discontinuities that mark the edges of the exposed circular zone. These boundary points are detected from the scan data and remapped to 0 mm and 150 mm respectively. Let $x_{L}$ and $x_{R}$ be the detected left and right edge positions in the preliminary scan coordinate. The remapped scan coordinate is given in Equation (11):

(11) $$x_{scan}=D\cdot \frac{x_{raw}-x_{L}}{x_{R}-x_{L}}$$

where D = 150 mm. This mapping intentionally leaves scan points outside the blasted area at $x_{scan}<0$ mm or $x_{scan}>150$ mm. In the final plots, this is useful because it shows both the validated diameter and the neighboring external region.

## 3. Results

The outcomes of the loading Cases A, B and C are presented in this section. Two main behaviors are reported: a conventional axisymmetric response and an anomalous response exhibiting counterintuitive behavior (CIB). For each case, the high-speed tracking sequence, the final profile overlaid on the 3D scan section cut, and the pointwise displacement histories are reported. The comparison shows that the same processing pipeline yields a consistent diametral measurement in all cases, while exposing the limits of the axisymmetric assumption when the plate response is not perfectly symmetric about the marked diameter.

**Figure 6:**
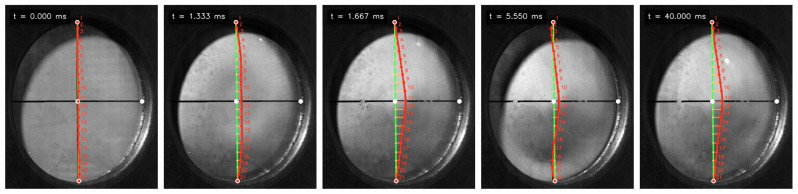
High-speed image sequence for Case A. The red tracked profile and numbered points show the displacement of the marked diameter from the first frame, before the arrival of the blast wave, to the last frame. Green lines show undeformed profile; red lines show deformed profile at variable times.

Figure 6 shows the high-speed tracking sequence for Case A. The initial frame shows the marked vertical diameter before deformation. After the blast arrival, the internal points move laterally while the edge anchors remain fixed. The deformed profile remains smooth and coherent throughout the event. The largest displacement occurs near the central region, and the displacement decreases toward the boundaries. This is the expected behavior for a clamped circular plate subjected to a uniform pressure pulse.

**Figure 7:**
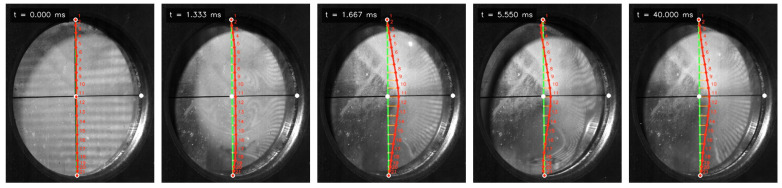
High-speed image sequence for Case B. The red tracked profile and numbered points show the displacement of the marked diameter from the first frame, before the arrival of the blast wave, to the last frame. Green lines show undeformed profile; red lines show deformed profile at variable times.

The same behavior is presented for Case B and shown in Figure 7.

**Figure 8:**
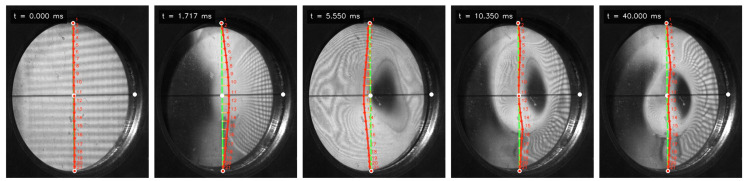
High-speed image sequence for Case C. The plate’s out-of-plane response is tracked following the red line. Different timeframes are presented showing the displacement vectors calculated at different points as they shift over time from their initial position, marked with the green line. Green lines show undeformed profile; red lines show deformed profile at variable times.

Figure 8 shows the high-speed tracking sequence for Case C. The initial frame shows the marked vertical diameter before deformation. The tracking follows the same procedure as in Case A. Initially, the plate deforms as expected: the tracked profile deflects outwards, with the central point moving away from the baseline in the direction opposite to the incoming blast.

At approximately 5 ms, however, the response diverges from the previous ones: the plate undergoes a dynamic snap-through and settles into an anomalous, non-axisymmetric permanent shape. The displaced region is no longer centered symmetrically about the marked diameter, and the peak deformation shifts away from the plate center towards an annular ring. This behavior is consistent with counterintuitive behavior (CIB), a well-documented dynamic instability in blast-loaded plates [8,10] associated with reversed snap-buckling. The onset of CIB is typically triggered by small perturbations in the loading symmetry or boundary conditions and may be further amplified by material imperfections: AA2024-T3 plates are manufactured by cold-rolling, which can introduce mild anisotropy and localized defects that provide the initial perturbation from which the non-axisymmetric mode grows. Despite the asymmetric final shape, the tracked diametral profile remains a valid one-dimensional measurement of the out-of-plane displacement along the marked line; however, it cannot be taken as representative of the full plate surface in this case. It is hypothesized that the cold-rolling process used to manufacture the AA2024-T3 sheet introduces a mild preferential orientation that could act as a localized symmetry-breaking perturbation and, under a nominally centered impulse, seed the reversed snap-buckling mode. It should be emphasized, however, that the present study does not include a dedicated characterization of the material anisotropy, local imperfections or actual boundary conditions; this explanation is therefore offered as an interpretative hypothesis rather than a demonstrated cause, and a slight loss of axisymmetry in the loading or clamping cannot be excluded as a contributing factor. A detailed investigation of the parameters governing the emergence of CIB is outside the scope of the present work; the interested reader is referred to [8,10].

**Figure 9:**
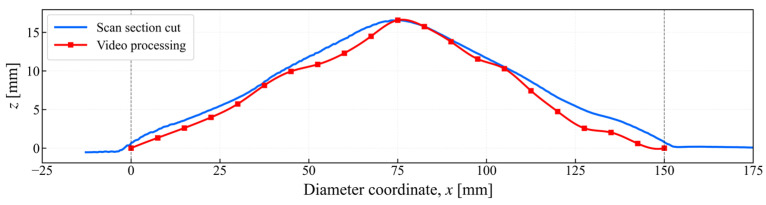
Final profile of Case A.

**Figure 10:**
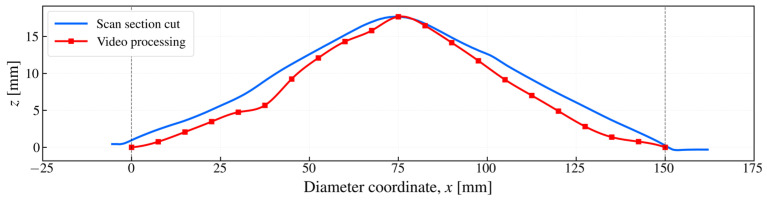
Final profile of Case B.

**Figure 11:**
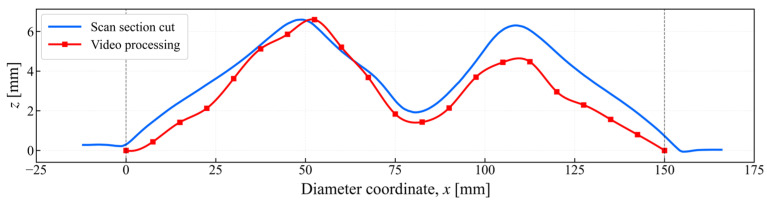
Final profile of Case C.

The final deformation profiles for the tests are presented in Figure 9, Figure 10 and Figure 11. These profiles are extracted from the tracking output by reading the displacement matrix at the last analyzed frame. The profiles are plotted in physical units after applying the rational pixel-to-mm mapping, so vertical displacements are expressed in millimeters, and horizontal positions correspond to the calibrated point locations along the marked diameter.

In all three cases the profiles are compared with the cross-section that is extracted from the post-test three-dimensional scan. The scan section is aligned to the same diameter as the marked line, so a direct spatial overlay is possible. Agreement between the video-derived profile and the scan profile provides a quantitative validation of the method output.

For Case A and B, respectively Figure 9 and Figure 10, the video-derived final profile agreed closely with the scan section cut. The peak displacement and the overall shape of the deformed diameter are captured accurately. Minor discrepancies at the boundary regions are attributable to the proximity of the edge anchors and to the limited spatial resolution of the point array near the clamped boundary.

For Case C (Figure 11), the video-derived final profile showed larger peak deformation than Cases A and B.

**Figure 12:**
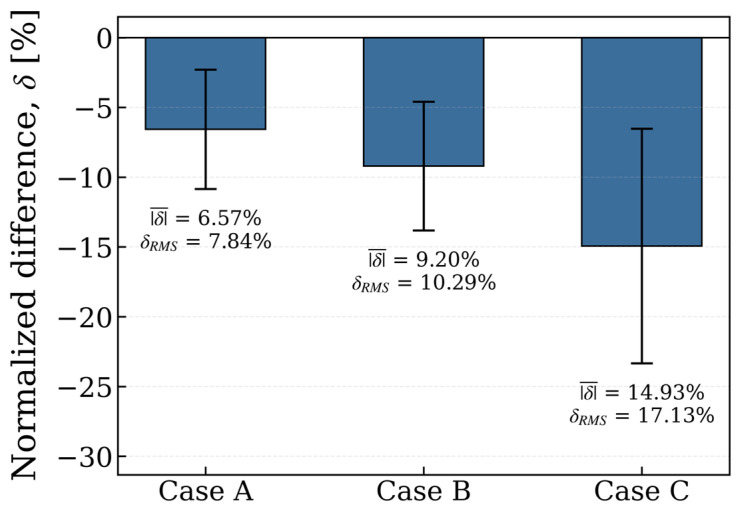
Normalized percentage residual δ between the video-based reconstruction and the reference 3D scan along the plate diameter, evaluated at internal points for Case A, Case B and Case C. Bars denote the mean normalized residual $\bar{\delta }$ and error bars the corresponding standard deviation; the mean absolute residual $\left|\bar{\delta }\right|$ and the normalized RMS residual $\delta_{RMS}$ are annotated for each case. Negative mean values indicate a slight systematic underestimation of displacement by the video-based method.

The agreement between the image-based reconstruction and the independent 3D scan was quantified by comparing the three final deformation profiles along the plate diameter. The scan-derived profile was treated as the reference, and comparison was restricted to internal points, excluding the two boundary points, which are more susceptible to edge-detection and coordinate-registration uncertainty. The local pointwise error e_i_ was defined as the difference between the video-derived displacement ${z_{v}}^{\mathrm{ed}}$ and the scan-derived displacement $z_{\mathrm{sa}}$ at each diametral coordinate $x_{i}$. Two absolute error indicators were computed: the root-mean-square error (RMSE) and the mean absolute error (MAE). A normalized percentage residual $\delta_{i}=100\cdot e_{i}/\Delta z_{\mathrm{sa}}$ was also computed, where $\Delta z_{\mathrm{sa}}$ is the total displacement range of the scan-derived profile; this normalization avoids instability near zero-displacement locations and expresses the mismatch as a fraction of the overall deformation amplitude. For Case A, the video profile required only a negligible horizontal registration correction of −0.042 mm. After alignment, the internal-point RMSE was 1.251 mm, the MAE was 1.048 mm, and the normalized indicators were $|\bar{\delta }|=6.57\%$ and $\delta_{RMS}=7.84\%$, confirming that the typical discrepancy remains below 8% of the total scan-measured deformation range. For Case B, a horizontal correction of 0.118 mm was required; after alignment the RMSE was 1.793 mm, the MAE was 1.604 mm and the normalized values were $|\bar{\delta }|=9.20\%$ and $\delta_{RMS}=10.29\%$. For Case C, a more substantial horizontal correction of 2.107 mm was required, reflecting the greater complexity of the anomalous deformation profile. After alignment the RMSE was 1.043 mm, the MAE was 0.909 mm, and the normalized values were $|\bar{\delta }|=14.93\%$ and $\delta_{RMS}=17.13\%.$ Three cases therefore demonstrate millimetric-level absolute agreement; the higher normalized residuals for Case C indicate that, although the absolute pointwise error is slightly smaller, the residuals represent a larger fraction of the total deformation amplitude. In all cases the signed mean normalized residual is negative, indicating a small systematic tendency for the video-derived profile to underestimate the scan-derived displacement. These considerations are graphically reported in Figure 12.

This result illustrates both the capability and the limitation of the method: the marked diameter is tracked consistently, but the underlying response of the plate in Case C is moderately violating the initial assumption of a pure axisymmetrical deformation pattern.

**Figure 13:**
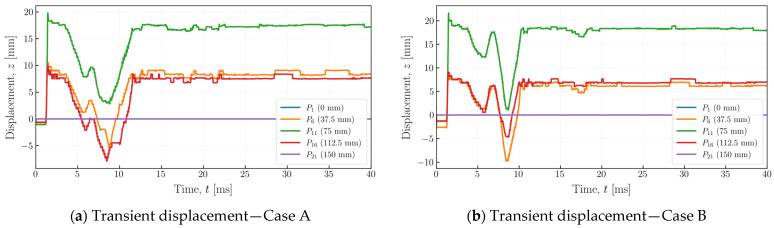
Displacement time histories of tracked points along the marked vertical diameter. Each curve corresponds to one of the 21 tracked points; colors indicate position from the plate center (largest displacement) to the clamped boundary (near-zero displacement). The histories are smooth and monotonic, consistent with a conventional axisymmetric blast response.—(**a**) Case A. (**b**) Case B.

**Figure 14:**
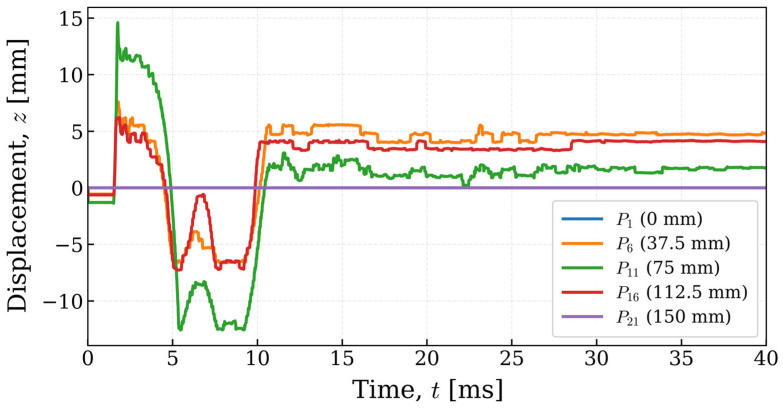
Displacement time histories of tracked points along the marked vertical diameter—Case C. Each curve corresponds to one of the 21 tracked points. Unlike Cases A and B, the histories exhibit an irregular, non-monotonic evolution after approximately 5 ms, and the final peak displacement is not located at the plate center, consistent with the counterintuitive behavior (CIB) response.

Figure 13 and Figure 14 show the displacement time histories extracted from the tracking matrix of individual points along the marked vertical line. Each curve corresponds to a single tracked point ranging from the middle-point to the plate’s boundaries. In Cases A and B, the histories are smooth and monotonic: the central points reach the largest displacements and the histories decay progressively toward both boundaries. In Case C, the final peak displacement is not located in the center of the plate due to the CIB.

**Figure 15:**
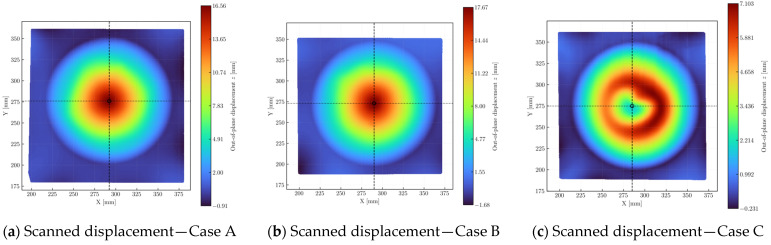
Post-test three-dimensional scan: out-of-plane displacement fields. (**a**) Case A: approximately axisymmetric displacement field with peak displacement concentrated at the center and a smooth decay toward the clamped boundary. (**b**) Case B: approximately axisymmetric displacement field with peak displacement concentrated at the center and a smooth decay toward the clamped boundary. (**c**) Case C: asymmetric displacement field showing a shift in the peak displacement from the geometric center and non-circular contours, interpreted as a departure from axisymmetry consistent with an instability-driven response (see Section 4.2).

Figure 15 shows the scanned displacement fields for all three tests. The displacement field is obtained from the post-test three-dimensional scan by computing the out-of-plane displacement relative to the undeformed plate geometry. In Cases A and B, the displacement field is approximately axisymmetric, with the maximum displacement concentrated near the center and smooth decay toward the clamped boundary. In Case C, the displacement field is mildly asymmetric: the region of maximum displacement is shifted from the geometric center, and the contours are quasi-circular. This spatial asymmetry is consistent with an instability-driven response in Case C, although the present data do not establish its cause.

## 4. Discussion

The results of Section 3 are discussed here on five levels: the geometric principle that makes a single-camera, single-diameter measurement quantitative (Section 4.1); the deformation mechanisms governing the conventional and anomalous responses (Section 4.2); the accuracy of the method relative to stereo-DIC (Section 4.3); its applicability to broader explosion-dynamics problems and to more complex media (Section 4.4); and, after summarizing the residual uncertainty (Section 4.5), its practical value and the cost–accuracy trade-off it entails (Section 4.6).

### 4.1. Geometric Principles of the Method

The proposed method manages to produce trustworthy results due to the dimensional reduction linked to the characteristics of the experimental setup as the plate supported by circular clamping system subjected to a uniform blast-load generated by an EDST is expected to deflect uniformly around the transverse axis passing through its center-point. If this assumption holds, a line along its surface crossing its middle-point contains enough information to reconstruct the principal deformation profile. The algorithm does not need to infer a full displacement field; it only needs to measure the evolution of one material line.

The core mathematical formulation relies on the signed normal projection. This turns a tracked image coordinate into a scalar offset relative to the undeformed baseline. The projective mapping then turns the scalar offset into millimeters. The entire pipeline is therefore interpretable: each output displacement can be traced back to a detected image point, a geometric projection, and a calibrated scalar conversion.

### 4.2. Interpretation of the Anomalous Case

Case C shows the limitation of the proposed method. It demonstrates that a symmetry-based processing can recover the displacement of the marked diameter but cannot guarantee that the same profile represents perfectly the entire plate. The anomalous physical deformation observed after the test indicates that the plate response includes features outside the ideal axisymmetric model. In this case, the method retains its value as a one-dimensional diagnostic tool along the marked diameter, but the scan section and specimen inspection become essential for interpretation.

In order to investigate the applicability of this method, two distinct scenarios are considered in this study. Cases A and B demonstrate the performance of the method under conventional conditions, whereas Case C illustrates its behavior and limitations under unconventional response. Together, they define the operational envelope of the proposed approach.

### 4.3. Accuracy Relative to DIC

DIC reconstructs the full three-dimensional surface and the in-plane strain field, whereas the present method reconstructs a single out-of-plane displacement profile along one diameter. For that single restricted quantity, the out-of-plane displacement sampled along one diameter, the accuracy is comparable; this should be read strictly as agreement on that scalar quantity, and not as a general methodological equivalence between the two techniques, since DIC additionally resolves the full surface field and, where applicable, the in-plane strains. Validated against the independent 3D scan, the single-camera reconstruction reproduced the permanent profile with a root-mean-square error of 1.251 mm for the conventional Case A (normalized mean residual 6.57%), 1.793 mm for Case B (9.20%) and 1.043 mm for the anomalous Case C (14.93%) over deflections ranging from about 5 to 16 mm. These figures are of the same order as the out-of-plane uncertainties reported for DIC on comparable blast-loaded aluminum plates [16,17], yet they are obtained with one camera instead of two, without a speckle pattern, and with a two-position calibration rather than a full stereo calibration. The systematic, slightly negative mean residual indicates a small consistent underestimation of displacement rather than random scatter and is therefore correctable. DIC remains the superior and necessary choice whenever the full surface, the in-plane strains, or non-axisymmetric and three-dimensional kinematics must be resolved. The proposed method should be read as complementary to DIC, a validated, low-cost option for the specific class of symmetry-dominated responses, and not as a general replacement. It should also be noted that this validation concerns the permanent (residual) profile recovered from the post-test 3D scan; the agreement therefore supports the accuracy of the reconstructed final deformation but does not, by itself, independently validate the transient displacement histories during the dynamic event, for which a time-resolved reference (e.g., synchronized stereo-DIC, laser displacement sensors or LVDTs) would be required.

### 4.4. Applicability and Extension to More Complex Media

The method generalizes beyond the present monolithic aluminum plates to any blast- or impact-driven problem in which the dominant deformation field possesses a known symmetry that reduces it to a small number of parametrized generators. For isotropic plates under centered, quasi-uniform loading a single marked diameter is sufficient. For more complex media, such as fiber-metal laminates, sandwich panels or layered composites, the single-line assumption holds only while the macroscopic response remains axisymmetric; local mechanisms such as core crushing, delamination or fiber fracture break this symmetry and cannot be inferred from one generator. In such cases the framework still serves as a one-dimensional diagnostic along a chosen generator and can be extended by (i) marking two or more orthogonal generators to detect and quantify departures from axisymmetry, or (ii) combining the single calibrated camera with a known modal/shape basis of the expected response. Genuinely three-dimensional or fragmenting responses remain the domain of stereo-DIC. The single-camera approach is therefore best understood as a low-cost measurement principle for symmetry-constrained problems rather than a universal full-field technique.

### 4.5. Sources of Uncertainty

The main uncertainty sources are manual initialization of the anchors and center; visibility and contrast of the marked line; motion blur during the early high-speed phase; interference between the vertical and horizontal marks near the center; local reflections from the metallic surface; uncertainty in the rational pixel-to-millimeter mapping; ambiguity in selecting the exact scan section corresponding to the marked line; and limited pixel resolution of the frames and departures from axisymmetry.

An approximate, order-of-magnitude assessment of the dominant contributions can be made even without a full propagation analysis. At the calibrated scale of about 0.67 mm/px, the finite image resolution combined with sub-pixel localization of the marked line (typically a fraction of a pixel) limits the per-point detection accuracy to roughly 0.1–0.3 mm, while the two-position projective calibration, whose secant scales are known to about 1%, propagates to a comparable sub-millimeter uncertainty over the ±20 mm working range. The selection of the scan section introduces a positional ambiguity of at most one station spacing (7.5 mm), which affects the comparison mainly where the profile gradient is steep. These algorithmic contributions are individually at or below the millimeter level and cannot by themselves account for the observed RMSE of 1.0–1.8 mm; the residual is therefore dominated by physical departures from axisymmetry, negligible for the conventional Cases A and B but significant for the anomalous Case C, which cannot be removed by image processing. This ranking identifies the loss of axisymmetry, rather than pixel-level noise or calibration, as the leading source of uncertainty whenever the response is not strictly axisymmetric, consistent with the small systematic underestimation observed in all three cases.

Some of these errors are algorithmic and can be reduced with better image processing. Others are physical and cannot be removed by processing. For example, if the specimen deforms asymmetrically, the marked diameter remains a valid measurement line, but it is no longer a complete representation of the plate surface.

### 4.6. Practical Value and Cost–Accuracy Trade-Off

The practical value of the method is that it gives quantitative deformation histories with a very small experimental burden. The required preparation is limited to marking the plate, recording it with one high-speed camera, and performing a semi-automatic tracking operation. The output is directly useful for validating finite-element models, especially models that predict displacement histories and residual diametral profiles.

The method is also attractive for repeated campaigns. Once the camera and mapping coefficients are fixed, multiple tests can be processed consistently. The scan validation can then be used selectively to check the final deformation and quantify the reliability of the video-derived profiles. It should be emphasized that the reduced experimental burden is obtained at a defined cost in measurement completeness rather than in pointwise accuracy. Along with the marked diameter the method reproduces the scanned permanent profile to within about 1 mm (RMSE 1.25 mm, below 8% of the deformation range for the conventional case), which is adequate for displacement-based model validation. Unlike stereo-DIC, however, it does not provide a full-field surface measurement, in-plane strain fields, or information off the marked line, and it is quantitatively reliable only while the response remains close to axisymmetric. The trade-off is thus a loss of spatial coverage and strain information, not of out-of-plane displacement accuracy, in exchange for an order-of-magnitude reduction in instrumentation cost and preparation effort. Finally, it should be acknowledged that the present validation rests on a limited number of cases, deliberately chosen to span the conventional and anomalous response regimes rather than to characterize repeatability under nominally identical conditions; the sensitivity of the procedure to experimental variability and its statistical robustness therefore remain to be established, and the validation reported here should accordingly be regarded as preliminary.

## 5. Conclusions

A symmetry-based, single-camera perspective correction method was developed and validated for the high-speed deformation analysis of clamped circular blast-loaded plates, using a single marked diameter and a calibrated rational projective mapping. From the three shock tube tests and their comparison with post-test 3D scans, the following conclusions are drawn:

1. By exploiting the axisymmetry enforced by the circular clamping and the centered shock tube load, a single high-speed camera tracking one marked diameter recovers quantitative transient displacement histories and permanent profiles; a rational mapping, derived from projective geometry and calibrated from only two out-of-plane positions, converts the apparent pixel offsets into physical out-of-plane displacements.
2. Validation against independent 3D scan section cuts demonstrates satisfactory accuracy (RMSE 1.25, 1.79 and 1.04 mm; normalized mean residual 6.6%, 9.2% and 14.9% for Cases A, B and C), comparable to the out-of-plane accuracy of stereo-DIC but obtained at a fraction of its experimental cost and complexity establishing the method as a complement to, not a replacement for, full-field techniques.
3. The conventional cases follow a Mode I, membrane-dominated ductile mechanism, whereas the anomalous case exhibits counterintuitive behavior: a reversed snap-buckling instability, tentatively attributed to cold-rolling imperfections, that displaces the peak deflection to an annular ring. This case also defines the operational envelope of the method: the marked diameter remains valid, but scan validation and physical inspection become necessary once axisymmetry is lost.
4. The applicability of the method is intentionally bounded: it is quantitatively reliable only for configurations that combine geometric symmetry, centered loading and a predominantly axisymmetric response. Once non-axisymmetric deformation develops, as in Case C, the marked diameter still yields a valid one-dimensional out-of-plane measurement, but it no longer represents the full displacement field of the plate, and independent scan or full-field validation becomes necessary. The present results, obtained on a limited set of cases spanning the conventional and anomalous regimes, should accordingly be regarded as a preliminary demonstration of feasibility rather than a complete statistical validation.
5. The approach is a low-cost, interpretable alternative for symmetry-constrained blast and impact experiments and can be extended to more complex media through multiple marked generators or a known modal basis. Future work will pursue a direct stereo-DIC comparison to independently validate the transient displacement histories, which the present post-test 3D scan comparison does not assess, the use of two orthogonal diameters to quantify departures from axisymmetry, and a formal uncertainty propagation of the projective mapping and tracking procedure.

## Figures and Tables

**Table 1:** Nominal physical and quasi-static mechanical properties of the AA2024-T3 aluminum alloy [23,24].

Property	Symbol	Value
Density	ρ	2770 kg/m^3^
Young’s modulus	E	73.1 GPa
Poisson’s ratio	ν	0.33
0.2% proof (yield) stress	R_p02_	345 MPa
Ultimate tensile strength	R_m_	483 MPa
Elongation at fracture	A	18%

**Table 2:** Tests used for validation of the image elaboration framework. Here, m PG-3 represents the mass of the detonated PG-3 plastic-explosive charge, expressed in grams.

Test	m PG-3 (g)	Outcome
A	3.7	conventional
B	3.9	conventional
C	2.0	anomalous

**Table 3:** Value of the correction for the case study under analysis.

Coefficient	a (mm/px)	b (1/px)
Value	0.66937	0.002655

**Figure A1:**
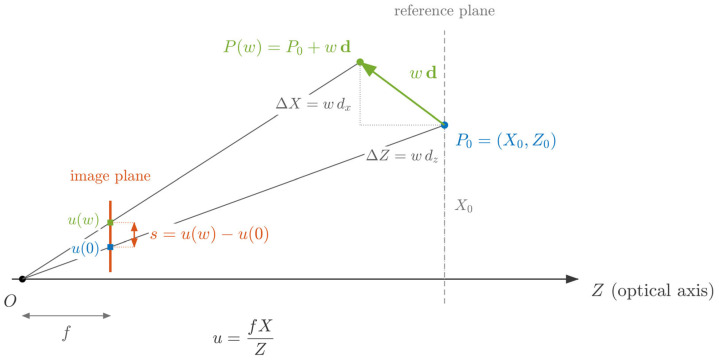
Geometry of the perspective projection. A physical out-of-plane displacement w along the oblique direction d produces both a depth change $\Delta Z=w\cdot d_{z}$ and a lateral shift $\Delta X=w\cdot d_{x}$, which the camera records as a single image-plane offset s=u(w)−u(0).

**Figure A2:**
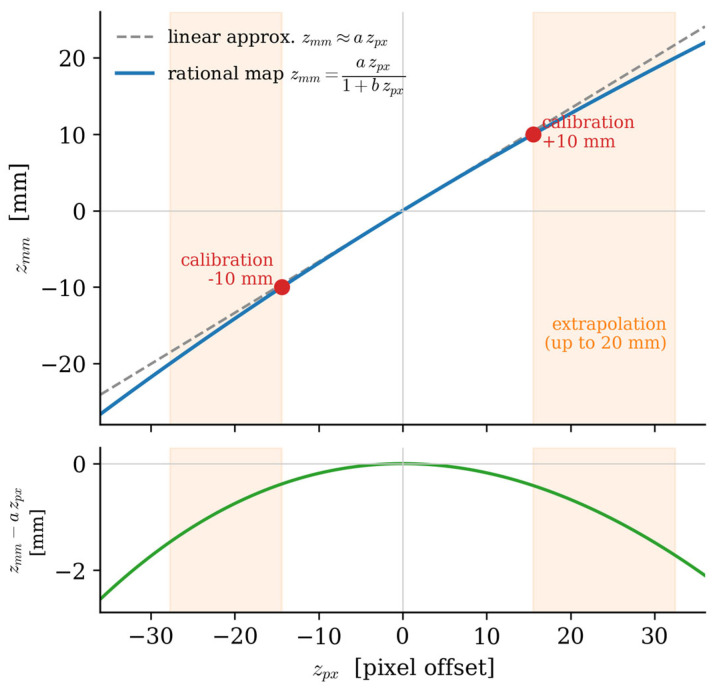
Rational pixel-to-millimeter mapping compared with its small offset linear approximation. The two calibration positions at ±10 mm fix the mapping over the considered range; the shaded bands mark the region up to ±20 mm. The lower panel reports the perspective correction.

**Figure A3:**
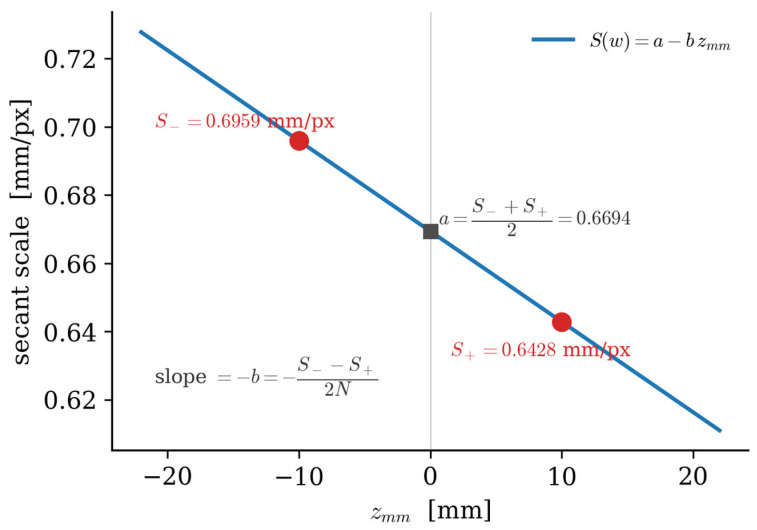
Secant scale S(w)=a−bw. The two calibration measurements $S_{-}$ and $S_{+}$ determine the intercept $a=(S\_+S_{+})/2$ and the slope $-b=-(S_{-}-S_{+})/(2N)$, illustrating that two points fully identify the two-parameter mapping.

## Data Availability

The original contributions presented in this study are included in the article. Further inquiries can be directed to the corresponding author.

## Abbreviations

The following abbreviations are used in this manuscript:

DIC	Digital Image Correlation
CIB	Counterintuitive Behavior
EDST	Explosion-Driven Shock Tube
RMSE	Root-Mean-Square Error
MAE	Mean Absolute Error

## Appendix A

This appendix provides the geometrical basis of the rational pixel-to-millimeter correction adopted in Equation (8). The purpose is to show that the proposed mapping is not an empirical interpolation law, but the one-dimensional form of the projective relation between the physical out-of-plane displacement of the plate and the corresponding apparent displacement measured in the image plane. The derivation starts with the pinhole camera model [12]. Let O be the optical center, Z the depth direction along the optical axis, X the lateral coordinate orthogonal to it, and f the focal length. A material point with coordinates (X, Z) is projected onto the image plane according to Equation (A1):

(A1) $$u=f\cdot \frac{X}{Z}$$

The non-linearity of the projection is therefore entirely associated with the perspective division by Z. This term is the origin of the rational form used in the correction. During the deformation, a point belonging to the marked line on the plate moves in space as a function of the physical out-of-plane displacement w, here corresponding to z_mm_. Assuming that the local trajectory can be approximated as rectilinear, the point position can be written as reported in Equation (A2),

(A2) $$P\left(w\right)=P_{0}+w\cdot d$$

where $P_{0}=(X_{0},Z_{0})$ is the undeformed position and $d=(d_{x},d_{z})$ is the direction of motion. Hence, as reported in Equation (A3),

(A3) $$X\left(w\right)=X_{0}+w\cdot d_{x};\;Z\left(w\right)=Z_{0}+w\cdot d_{z}.$$

The camera is not aligned with the displacement direction. Therefore, an out-of-plane motion produces both a change in depth, $\Delta Z=w\cdot d_{z}$, and a lateral shift, $\Delta X=w\cdot d_{x}$. This oblique observation geometry is precisely what makes the out-of-plane displacement visible as an in-plane image displacement, as illustrated schematically in Figure A1.

The apparent image-plane offset is defined as the difference between the projected position of the deformed point and that of the undeformed point (Equation (A4)):

(A4) $$s\left(w\right)=u\left(w\right)-u\left(0\right)=f\cdot \frac{X_{0}+wd_{x}}{Z_{0}+wd_{z}}-f\cdot \frac{X_{0}}{Z_{0}}.$$

Introducing a common denominator leads to Equation (A5):

(A5) $$s\left(w\right)=f\cdot \frac{Z_{0}\left(X_{0}+wd_{x}\right)-X_{0}\left(Z_{0}+wd_{z}\right)}{Z_{0}\left(Z_{0}+wd_{z}\right)}=f\cdot \frac{w(Z_{0}d_{x}-X_{0}d_{z})}{Z_{0}\left(Z_{0}+wd_{z}\right)}$$

By factoring the denominator, Equation (A5) can be written as Equation (A6).

(A6) $$s\left(w\right)=\frac{\alpha w}{1+\beta w};\;\;\alpha =f\cdot \frac{Z_{0}d_{x}-X_{0}d_{z}}{Z_{0}^{2}};\;\beta =\frac{d_{z}}{Z_{0}}$$

Equation (A6) is a linear-fractional, or Möbius, function of the physical displacement. Its zero constant term follows directly from the definition of the offset, since s(0)=0. The coefficient α represents the small-displacement sensitivity, while β accounts for the curvature introduced by perspective effects. Therefore, the rational form is not arbitrarily selected as a fitting function; it follows directly from the projective nature of the camera model.

Since the experimental procedure measures the image offset and requires the physical displacement, Equation (A6) is inverted in Equation (A7). The inverse of a Möbius function is again a Möbius function:

(A7) $$w=\frac{s}{\alpha -\beta \cdot s}$$

When the image-plane offset is expressed in pixels, the proportionality between physical image-plane distance and pixel displacement can be absorbed into the coefficients. With the sign convention used, this gives the working correction law as reported in Equation (A8).

(A8) $$z_{mm}=\frac{az_{px}}{1+bz_{px}}$$

where z_px_ is the measured pixel offset, and a and b are two scalar calibration parameters. These two constants collect the effect of the focal length, pixel pitch, initial distance from the camera, and oblique viewing geometry. As a result, the complete optical configuration does not need to be known explicitly.

The need for only two calibration positions can be understood from the degrees of freedom of the mapping. A general one-dimensional Möbius transformation, as reported in Equation (A9),

(A9) $$y=\frac{Ax+B}{Cx+D}$$

has three independent degrees of freedom, since multiplication of all coefficients by the same scalar leaves the mapping unchanged. In the present case, the origin is fixed by construction: a zero physical displacement must correspond to a zero-pixel offset. This imposes B=0, reducing the transformation to two independent parameters. Consequently, two known displacement positions are sufficient to identify the full mapping.

The relationship between the calibration coefficients and the measured scales can be obtained directly from Equation (A8). Rearranging, it is possible to obtain Equation (A10).

(A10) $$\frac{z_{mm}}{z_{px}}=a-bz_{mm}$$

The secant scale is thus reported in Equation (A11):

(A11) $$s\left(w\right)=a-bw.$$

For two symmetric calibration positions w=±N, with N=10 mm, it is possible to obtain Equation (A12):

(A12) $$S_{+}=a-Nb;\;\;S_{-}=a+Nb.$$

And solving the linear system gives the coefficients reported in Equation (A13):

(A13) $$a=\frac{S_{-}+S_{+}}{2};\;\;\;b=\frac{S_{-}-S_{+}}{2N}.$$

Using the measured calibration scales $S_{-}$ = 0.6959 mm/px and $S_{+}$ = 0.6428 mm/px, the resulting coefficients are a = 0.66937 mm/px and b = 0.002655 px^−1^. The positive sign of b follows from the adopted sign convention and corresponds to the target approaching the camera during positive displacement.

Figure A2 compares the rational correction with its small offset linear approximation. Close to the origin, the two mappings are interchangeable because the denominator in Equation (8) remains close to unity. At larger offsets, however, the rational term accounts for the perspective-induced variation in scale.

Figure A3 further illustrates that the two calibration measurements determine the intercept and slope of the secant-scale relation, and therefore fully define the two-parameter mapping.

In summary, the denominator $1+bz_{px}$ represents the perspective division 1/Z expressed in pixel coordinates. The two-parameter form arises because the origin is fixed by the undeformed configuration, leaving only two degrees of freedom in the projective mapping.

## References

[1] Nurick G.N., Martin J.B. (1989). Deformation of thin plates subjected to impulsive loading—A review. Part I: Theoretical considerations. Int. J. Impact Eng..

[2] Nurick G.N., Martin J.B. (1989). Deformation of thin plates subjected to impulsive loading—A review. Part II: Experimental studies. Int. J. Impact Eng..

[3] Teeling-Smith R.G., Nurick G.N. (1991). The deformation and tearing of thin circular plates subjected to impulsive loads. Int. J. Impact Eng..

[4] Wen H.M. (1998). Deformation and tearing of clamped circular work-hardening plates under impulsive loading. Int. J. Press. Vessel. Pip..

[5] Chung Kim Yuen S., Nurick G.N., Langdon G.S., Iyer Y. (2017). Deformation of thin plates subjected to impulsive load: Part III—An update 25 years on. Int. J. Impact Eng..

[6] Louar M.A., Belkassem B., Ousji H., Spranghers K., Kakogiannis D., Pyl L., Vantomme J. (2015). Explosive driven shock tube loading of aluminium plates: Experimental study. Int. J. Impact Eng..

[7] Aune V., Fagerholt E., Hauge K.O., Langseth M., Børvik T. (2016). Experimental study on the response of thin aluminium and steel plates subjected to airblast loading. Int. J. Impact Eng..

[8] Aune V., Valsamos G., Casadei F., Larcher M., Langseth M., Børvik T. (2017). Numerical study on the structural response of blast-loaded thin aluminium and steel plates. Int. J. Impact Eng..

[9] Granum H., Aune V., Børvik T., Hopperstad O.S. (2018). Aluminium plates with pre-formed slits subjected to blast loading. EPJ Web Conf..

[10] Xu Z., Liu Y., Shi Z., Huang F. (2022). Typical characteristics of counterintuitive behavior in thin aluminum plates under blast loading. Int. J. Impact Eng..

[11] Gargano A., Mouritz A.P. (2023). Comparative study of the explosive blast resistance of metal and composite materials used in defence platforms. Compos. Part C Open Access.

[12] Ben Rhouma M., Maazoun A., Aminou A., Belkassem B., Vandenbruwane I., Tysmans T., Lecompte D. (2024). Blast loading of small-scale circular RC columns using an explosive-driven shock tube. Buildings.

[13] Mottahedi A., Aziz N., Remennikov A., Mirzaghorbanali A. (2026). Pull-out capacity and energy absorption of cable bolts under impact loading. Int. J. Min. Sci. Technol..

[14] Pan B., Qian K., Xie H., Asundi A. (2009). Two-dimensional digital image correlation for in-plane displacement and strain measurement: A review. Meas. Sci. Technol..

[15] Sutton M.A., Orteu J.-J., Schreier H.W. (2009). Image Correlation for Shape, Motion and Deformation Measurements: Basic Concepts, Theory and Applications.

[16] Tiwari V., Sutton M.A., McNeill S.R., Xu S., Deng X., Fourney W.L., Bretall D. (2009). Application of 3D image correlation for full-field transient plate deformation measurements during blast loading. Int. J. Impact Eng..

[17] Spranghers K., Vasilakos I., Lecompte D., Sol H., Vantomme J. (2012). Full-field deformation measurements of aluminum plates under free air blast loading. Exp. Mech..

[18] Spranghers K., Vasilakos I., Lecompte D., Sol H., Vantomme J. (2013). Numerical simulation and experimental validation of the dynamic response of aluminum plates under free air explosions. Int. J. Impact Eng..

[19] Atoui O., Maazoun A., Aminou A., Belkassem B., Pyl L., Lecompte D. (2023). Dynamic behavior of aluminum plates subjected to sequential fragment impact and blast loading: An experimental study. Appl. Sci..

[20] Hartley R., Zisserman A. (2003). Multiple View Geometry in Computer Vision.

[21] Zhang Z. (2000). A flexible new technique for camera calibration. IEEE Trans. Pattern Anal. Mach. Intell..

[22] Heikkilä J. (2000). Geometric camera calibration using circular control points. IEEE Trans. Pattern Anal. Mach. Intell..

[23] Buyuk M., Kan S., Loikkanen M.J. (2009). Explicit finite-element analysis of 2024-T3/T351 aluminum material under impact loading for airplane engine containment and fragment shielding. J. Aerosp. Eng..

[24] ASM International Handbook Committee (1990). ASM Handbook, Volume 2: Properties and Selection: Nonferrous Alloys and Special-Purpose Materials.

